# Influence of the Tertile of Birth on Anthropometric Variables, Anaerobic Parameters and Quantitative Muscle Ultrasound in School Children

**DOI:** 10.3390/ijerph18137083

**Published:** 2021-07-02

**Authors:** Juan Carlos Giraldo García, Elena Hernández-Hernández

**Affiliations:** 1 Área Biomédica, GESTAS Research Group, Politécnico Colombiano Jaime Isaza Cadavid, Medellín 050021, Colombia; 2 Sport and Computer Science Department, Universidad Pablo de Olavide, 41013 Seville, Spain; 3 SEJ-570 MOTIVA2 Research Group, Universidad Pablo de Olavide, 41013 Seville, Spain

**Keywords:** children, ultrasound, muscular development, quadriceps muscle, relative age effect, sport

## Abstract

The relative age effect (RAE) has been studied and demonstrated in the literature. Our study evaluated the influence of birth tertile on anthropometric variables, anaerobic parameters, and quantitative muscle ultrasound in school children. A transversal, comparative, non-randomized study was conducted with 159 participants (9.36 ± 0.84 years) recruited by purposive sampling, of whom 70 were girls (9.50 ± 0.77 years) and 89 were boys (9.25 ± 0.88 years). The sample was divided into groups based on the year of birth, and each group was divided into tertiles. The anthropometric parameters of body weight, height, and fat percentage were measured, and then a right quadriceps ultrasound was performed, followed by the evaluation of CMJ and continuous jumps. Comparison of tertile subgroups showed significant differences in the vertical jump, in girls (CMJ, PCMJ, PCMJR, PCMJDE, PCMJDER, and PP15) and in boys (PCMJ, PCMJR, PCMJDE, and PP15). The results indicate that being born in the first months of the year may have a positive influence on performance in anaerobic tests, such as vertical jump, and on the quantitative ultrasound results of the quadriceps.

## 1. Introduction

The relative age effect (RAE) has been studied and demonstrated in the scientific literature [1,2]. Studies conducted on this topic have shown that people born in dates close to the beginning of January have certain advantages in terms of performance, both in sports [1] and in academically [3,4]. This becomes important for the selection of sport talents based on the result of certain physical and/or motor tests obtained by athletes of the same chronological age. An example of this is found in a 2016 study performed by Müller et al. [5] in under-9 football players, who competed in the Vienna European football tournament. These authors showed that the probability of a football player to be selected for a European championship was 2.7 to 4.9 times greater for those born in the first quarter of the year compared to those born in the other three quarters [5]. As in the mentioned study, another investigation, conducted among Italian under-15 players and older (including Serie A), reported that the players born closer to the cut-off date were more selectable due to their anthropometric development and their greater strength levels and specific resistance compared to those born at later dates [6], which granted them better results in the applied tests. Moreover, some studies have shown the presence of a greater number of competitors in different sports born in the first trimester of the year compared to the second trimester of the same category based on age [3,7,8,9]. This has been observed in both individual and team sports, and also in both sexes [7]. As a consequence of this selection at early ages, it could be inferred that the divides are greater at these ages and become narrower toward adulthood; however, these divides have been shown to be irrecoverable [10].

Vertical jump height has a practical value in the evaluation of anaerobic power [11]. Parameters of vertical jump and leg power are used by coaches and sport professionals to distinguish among individuals of different performance levels [12], and jump is one of the main ways of evaluating anaerobic metabolism. CMJ (counter-movement jump) is a widely used test to measure performance in talent detection and follow-up, training control, and even development analysis [13]. A study conducted in Germany, which assessed 1835 children (4 to 17 years of age) with CMJ, concluded that peak strength does not seem to influence jump performance directly, as it did not change significantly with the increase of age, whereas the height of the jump did show a significant difference [13]. Studies that evaluated RAE in adolescents through vertical jump have not reported statistically significant differences [2,14,15].

The difference between males and females in the capacity to produce power during the jump can be primarily explained in terms of differences in muscular parameters, particularly in the muscle volume of the quadriceps femoris [16]. Ultrasound is a useful tool to measure the cross-sectional area (CSA) of the muscle accurately, and this is strongly correlated with its volume [17]. In a longitudinal study carried out in children over a period of four years, in which muscle strength and ultrasound measures were evaluated, muscle strength and thickness showed clinically relevant modifications, influenced by changes in weight, height, and age [18]. Unlike in the previous case, a recent study has shown significant correlations between muscle thickness and EI (echo-intensity) in the vastus lateralis and rectus femoris using some measurements of athletic performance and isometric strength in 12-year-old children [19]. These positive results, obtained in other age groups, have motivated the possibility of using muscle thickness and EI (still under research) [20] as indicators for some strength parameters among school children. It would be possible to develop a non-invasive tool, applicable to children, to predict aspects related to the amount of muscle that influence the levels of muscle power. Due to its safe character, ultrasound is an excellent method to evaluate and monitor muscle mass, both in quantity and quality, as well as the changes produced by training or by the natural development of the individual. There are studies on the RAE that used anthropometric variables or physical and motor tests [2,15]; however, to the best of our knowledge, the RAE has not been studied using quantitative ultrasound as a tool to evaluate the physiological aspects of muscle groups of the lower limbs in children and, particularly, in school children. Therefore, the present study evaluated the influence of birth tertile on anthropometric variables, anaerobic parameters, and quantitative muscle ultrasound in school children.

## 2. Materials and Methods

### 2.1. Participants

A total of 159 school children (9.36 ± 0.84 years), of whom 70 were girls (9.50 ± 0.77 years) and 89 were boys (9.25 ± 0.88 years), were recruited by purposive sampling from two sports initiation schools and one primary school in Medellin city (Colombia). The participants carried out two 45-min weekly sessions of physical education and, on Saturdays, they attended a 3-h sports initiation class, in which different sports modalities were practiced with intensity fluctuations, such as football, volleyball, basketball, swimming, and gymnastics. The exclusion criteria were: presence of cardiovascular or metabolic disease, musculoskeletal injuries, or Tanner self-reported sexual development different from Stage 1 [21]. A total of 192 minors attended the meeting, of whom 6 were excluded for having a Tanner score different from 1, another minor was excluded for presenting a diagnosis of diabetes mellitus, and another was excluded for neurological disease. The total evaluation was carried out on 184 minors, of whom those born in 2007 and 2011 were excluded, as the resulting groups for these years of birth were too small.

The participants and their parents signed an informed assent and consent, respectively. The study protocol was approved by the Ethics Committee of the Jaime Isaza Cadavid Polytechnic University (approval number: 21214001-201701009536) following the guidelines of the Declaration of Helsinki. The sample was divided by year of birth: 2007, 2008, 2009, 2010, and 2011.

### 2.2. Design

A cross-sectional, comparative, non-randomized study was conducted between October and December 2018. Information about the study was given by the instructors of the sports initiation schools to the potential participants, who were encouraged to participate. Potential participants were called for a meeting and, after explaining the project again to both the minors and their parents, participants were invited to sign the informed assent and consent. The target study population consisted of 266 minors. All participants attended the laboratory of Jaime Isaza Cadavid Polytechnic University or a space adapted to serve as a laboratory in the facilities of San José de Las Vegas and Lucrecio Jaramillo Schools; all of these buildings are located in the city of Medellin (Colombia). The following anthropometric measurements were recorded in these facilities: body weight, height, and fat percentage. The right quadriceps ultrasound was then performed, followed by the evaluation of jumps, beginning with CMJ and concluding with continuous jumps. The study variables are described below.

### 2.3. Age

The sample was divided into groups based on year of birth. The individuals born in 2007 (*n* = 4) and 2011 (*n* = 21) were excluded from the statistical analysis, due to the small number of research units obtained after dividing them into subgroups. Each year was divided by tertiles [22,23], because the groups were too small for division by quartiles. The first third (GE1) consisted of participants born between 1st January and 30th April. The second third (GE2) comprised those born between 1st May and 31st August. Lastly, the final third included those born between 1st September and 31st December.

### 2.4. Quantitative Ultrasound

Cross-sectional and longitudinal images of the quadriceps femoris of the right leg were obtained using a mode B device (B-Ultrasonic Diagnostic System, Contec, CMS600P2, Hubei, China). A linear transducer (gain: 58, frequency: 7.5 MHz; depth: 6 cm), covered with a water-soluble transmitter gel (in sufficient amount to prevent the compression of the skin surface), was placed perpendicular to the longitudinal and cross-sectional axes of the quadriceps femoris at the middle point between the anterior superior iliac spine and the upper pole, and between the latter and the supero-external angle of the knee for the anterior and lateral images, respectively. The participants were assessed in the supine position and were asked not to perform any vigorous physical effort the day before the evaluation. For each middle point, two longitudinal and two cross-sectional images were taken. The frozen image was digitalized and subsequently analyzed using the open-software ImageJ (National Institute of Health, Bethesda, Montgomery, Maryland, MD, USA, version IJ 1.46).

The anterior cross-sectional images were used to measure the muscle thickness of the rectus femoris (from the lower margin of the anterior fascia of the rectus femoris to the upper margin of the posterior fascia of the rectus femoris); the thickness of the vastus intermedius (lower margin of the intermuscular fascia and the periosteum of the femur); and the total thickness of the anterior quadriceps (from the lower margin of the rectus femoris to the periosteum of the femur) [24,25]. The lateral cross-sectional images were used to measure the thickness of the vastus externus (from the lower margin of the anterior fascia of the vastus externus to the upper margin of the posterior fascia of the vastus externus); the thickness of the vastus intermedius in lateral view (lower margin of the intermuscular fascia and the periosteum of the femur); and the total thickness of the lateral quadriceps (from the lower margin of the vastus externus to periosteum of the femur). The cross-sectional images were also used to determine the EI of the different muscles evaluated using the histogram tool in ImageJ.

**Figure 1:**
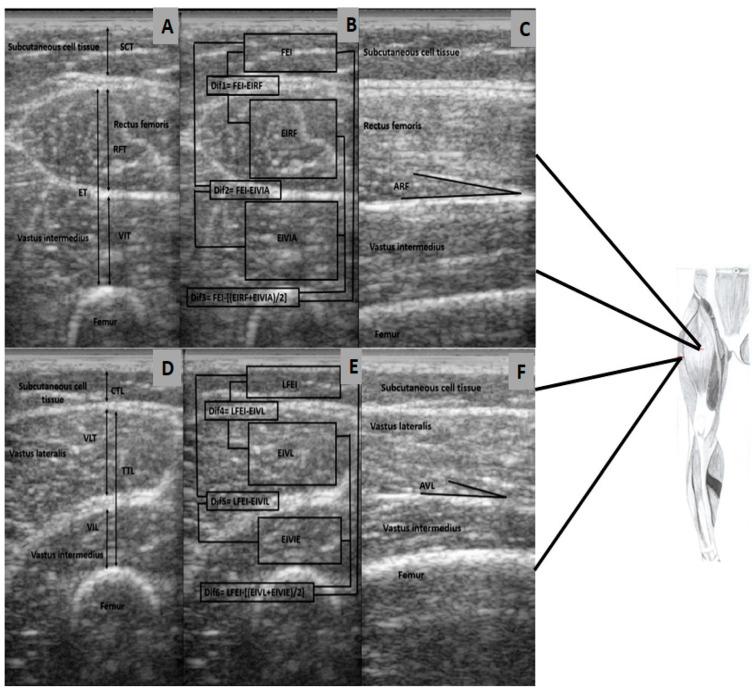
Image of the anterior and lateral regions of the thigh. (**A**) Transversal section, measurement of thickness. (**B**) Transversal section, areas of interest for the measurement of eco-intensity, the differences of EI with subcutaneous adipose tissue (Dif1 to Dif3) and the % of muscle fat. (**C**) Longitudinal section and measurement of the pennation angle. (**D**) Transversal section, measurement of thickness. (**E**) Transversal section, areas of interest for the measurement of eco-intensity, the differences of EI with subcutaneous adipose tissue (Dif4–Dif6) and the % of muscle fat. (**F**) Longitudinal section, measurement of the pennation angle. EVIE: VI thickness in the vastus externus, ETL: total thickness of the vastus externus, AVL: pennation angle of vastus lateralis.

The region of interest was selected as the largest rectangular area of each muscle without including fascia. The mean value of the two images was expressed as a value between 0 (black) and 255 (white). EI was corrected with subcutaneous adipose tissue (SAT) thickness, as proposed by Young et al., and fat percentage was measured with the method proposed by the same authors for all muscles [26]. Moreover, as a control strategy, we measured the difference of the EI of the fat with respect to each portion of the evaluated quadriceps (Dif1–Dif6) [27]. The cross-sectional images were used to determine the pennation angle of the rectus femoris and vastus externus (Figure 1). The values used for the statistical analysis of muscle thickness and pennation angle were the mean values of the two measurements of each image. The coefficient of variation of the two measurements taken at different moments, on the same day, from 10 individuals was 5.0% for muscle thickness, 0.4% for EI, and 0.8% for pennation angle. The ultrasound was conducted by a physician with experience in musculoskeletal ultrasound. The results were analyzed by two evaluators.

### 2.5. Anthropometry

Body weight and height were measured with the participants barefoot and in sports clothing. The body fat percentage was estimated according to Lohman’s skinfolds, measured in two different places: triceps and subscapularis muscle [28]. The measurements were recorded by students in the last semester of Physical Education with level 1 Anthropology, applying the ISAK procedures. Weight was expressed in kilograms and measured using a Detecto scale (DET 339, Detecto, Bogotá, Colombia). Height was measured in meters using a Kramer height rod (Kramer, Bogotá, Colombia). Fat percentage was recorded with a Slim Guide adipometer (Creative Health Products, Miami, FL, USA).

### 2.6. Vertical Jump

Explosive strength was evaluated through CMJ and repeated jumps (RJ15). At the beginning of the session, all participants performed a general dynamic warm-up, finishing with the execution of six jumps, with a progressive level of effort. The CMJ was conducted three times, and the best jump was used for the statistical analysis. After two minutes of recovery, they carried out the RJ15, which consisted in consecutive CMJs for 15 s. Throughout the test, the children were verbally stimulated. To ensure the correct execution of each jump, they were evaluated using a checklist, which allowed verification that each jump met the key aspects for correct execution. If the execution was incorrect, the participant was given 3 min of rest, and then the jump was repeated. The jumps that did not meet the acceptance criteria were considered null. With the information obtained from both jumps (CMJ and RJ15), the power of the vertical jump (CMJ) and the proportion of fast-twitch (FT) fibers (RJ15) were calculated.

The jumps were measured with an AXON JUMP^®^ mat (Axon Bioengeniería Deportiva, Buenos Aires, Argentina), using the Axon Jump 4.0 software, which measured the flight time and, in the case of RJ15, the contact time. For all jumps, the participants were requested to keep their hands on their waist. The RJ15 was conducted to calculate the mean power (PP = g2∗Tf∗15/4n(15 − Tf)) and the FT-fiber distribution % (%FT = 48.31 + (g2∗Tf∗15)/1.04n(15 − Tf) [29]. CMJ power was obtained with Sayers’s formula (CMJpower (W) = (51.9∗CMJ height(cm)) + (48.9∗body weight(Kg)) − 2007) [30]. CMJ power by thrust distance was obtained with the formula proposed by Jiménez-Reyes et al. (P = mg((h/hpo2) + 1) √gh/2) [31].

### 2.7. Statistical Analysis

For the descriptive analysis of the demographic, anthropometric, ultrasound, and functional data, we used absolute and relative frequencies and summary indicators such as median and median absolute deviation (MAD). To compare the relative age groups with the anthropometric, ultrasound, and functional indicators in girls and boys, the Kruskal–Wallis test was used, complemented with the epsilon squared rank coefficient as a measure of the effect size. To correlate age and the functional and ultrasound indicators in both girls and boys, we applied the Spearman’s correlation coefficient, represented in a color intensity matrix. To determine the effect size (ES), the following classification was used: small (S) = 0.01 to <0.08, medium (M) = 0.08 to <0.26, and large (L) ≥ 0.26 [32].

## 3. Results

In the comparison between the birth tertile by sex, significant differences were found, in girls in body weight (2009, *p* = 0.02; ES = 0.23) and height (2009, *p* = 0.004; ES = 0.33), and, in boys in body weight (2008, *p* = 0.023; ES = 0.314; 2009, *p* = 0.032; ES = 0.215), height (2008, *p* = 0.003; ES = 0.498), and BMI (2009, *p* = 0.04; ES = 0.194). No significant differences were found in fat percentage. The results are shown in Table 1.

Regarding the vertical jump variables, a comparison of the subgroups showed significant differences in girls in CMJ (2009, *p* = 0.001; ES = 0.413); PCMJ (2009, *p* = 0.001; ES = 0.406); PCMJR (2009, *p* ≤ 0.001; ES = 0.545); PCMJDE (2009, *p* ≤ 0.001; ES = 0.469), PCMJDER (2009, *p* = 0.046; ES = 0.186); and PP15 (2009, *p* = 0.023; ES = 0.227). Significant differences were found in boys in PCMJ (2008, *p* = 0.044; ES = 0.26; 2009, *p* = 0.006; ES = 0.221); PCMJR (2009, *p* = 0.013; ES = 0.27); PCMJDE (2009, *p* = 0.03; ES = 0.219); and PP15 (2009, *p* = 0.025; ES = 0.23). No significant differences were found in those born in the year 2010. The results are presented in Table 2.

Regarding the ultrasound variables, a comparison of the subgroups showed significant differences in girls in AVL (2009, *p* = 0.033; ES = 0.207); PGRF (2010, *p* = 0.02; ES = 0.518); DIF1C (2010, *p* = 0.023; ES = 0.503; and Dif4C (2010, *p* = 0.049; ES = 0.401). In boys, significant differences were found in EVIE (2009, *p* = 0.026; ES = 0.228); ETL (2009, *p* = 0.044; ES = 0.195); EIVIC (2010, *p* = 0.033; ES = 0.228); and PGVI (2010, *p* = 0.033; ES = 0.228). No significant differences were found in those born in the year 2008. The results are presented in Table 3.

**Figure 2:**
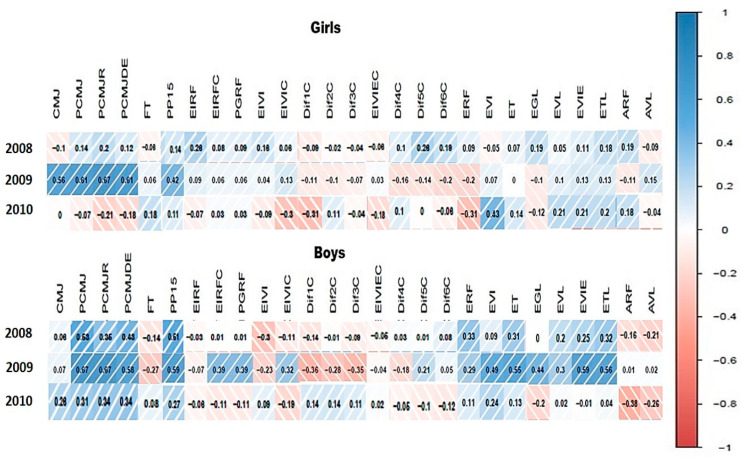
Correlations between age and the vertical jump and ultrasound variables.

As can be observed in Figure 2, the vertical jump variables CMJ, PCMJ, PCMJR, PCMJDE, and PP15 are moderately correlated with age (0.42–0.67) in both girls and boys. The EI ultrasound variables EIRFC, PGRF, EIVIC (0.32–0.39), Dif1C, Dif2C, and Dif3C (−0.35 and −0.36), and thickness and pennation angle ERF, EVI, ET, EGL, EVL, EVIE, and ETL (0.29–0.59) are also moderately correlated with age. The intensity of the blue color shows a greater positive correlation, whereas the intensity of the red color shows a greater negative correlation.

## 4. Discussion

The main objective of this study was to evaluate the influence of birth tertile on anthropometric variables, anaerobic parameters, and quantitative muscle ultrasound in school children. The analysis of the results showed significant differences in the anthropometric variables in several subgroups, specifically in body weight, height, and skinfolds. Similarly, the variables of vertical jump revealed differences in both girls and boys, as did the ultrasound variables of the quadriceps. Previous studies have reported that RAE appears particularly during adolescence, when the differences in physical attributes are more pronounced [2]. As is shown in this study, the RAE for body weight, height, muscle power, and vertical jump in adolescents [2,33] could indicate an advance of RAE in school children. Specifically, variables such as body weight, height, and vertical jump measures could be considered physical performance selection measurements that favor individuals born in the first third of the year.

Similarly, significant differences were found in numerous parameters of anaerobic power measured through vertical jump—in both girls and boys—that favored individuals born in the first third of the year. Similar results have been reported in other studies that evaluated differences in physical profile, specifically in speed tests of 10 and 30 m and in SJ (Squat Jump), which appeared with and without the inclusion of bone age in the analysis [34]. This highlights the importance of monitoring sport development in the long term, avoiding the selection of children based merely on the obvious advantages in the physical profile, and discarding those of a lower profile, especially in athletic sports where the RAE is larger. Long-term monitoring prevents the phenomenon that occurs in education known as “the Matthew” effect—that is, providing better training stimulation to those with a better physical profile and denying the same possibilities to those with a worse profile [35]. However, other studies have not reported differences in vertical jump or other physical measures, although this could be due to the fact that their samples were composed of adolescents [36,37]. Anaerobic power improves significantly from the age of 6 years to 12 years, when the levels of testosterone remain unaltered [38], thus older ages may be too late for talent detection.

There was a positive correlation between vertical jump power and age in each of the analyzed years, demonstrating that the differences in anaerobic power measured through vertical jump increased with the growth and development of the minor, and such differences were evidenced in several time points and periods of time even as short as less than one year of difference. This implies that minimal differences in age, even between months of the same year of birth, are obvious at the muscle level; therefore, the greater the difference in months, the greater the RAE. These correlations were constant in the three age groups evaluated in boys, whereas the girls only showed significant correlations in the 2009 group, which is in line with prior studies that have reported a greater RAE in males than females [39]. However, it is important to consider, again, that the statistical power that resulted from the larger number of research units among girls born in 2009 suggests a significant correlation that the other two groups (2008 and 2010) did not reach due to their smaller number of research units. With this in mind, the age correlation in those born in 2009, both girls and boys, is in line with multiple studies which report that jump height doubles between the age of 5 years and 13 years similarly in both sexes [29].

The diameter of the human muscle fiber increases with age, and the clear sex differences in this diameter appear around the age of 10 years [40]. This could explain the positive correlation between age and muscle thickness measured by ultrasound, which was more constant in boys in the three years evaluated than in girls. This is in agreement with studies that have demonstrated a strong association between muscle mass and muscle strength during growth [41].

Age was significantly correlated with the measures of ultrasound EI, particularly in the 2009 group, which could be explained by the larger quantity of data analyzed from that year. EI is a way to evaluate muscle quality; finding significant correlations in age for the same year of birth shows the evolution that muscles undergo intervals in which the changes, especially physical changes, flatten with respect to age periods in which they appear more rapidly, as is the case of pre-school and puberty. However, although such changes are slower, they are also important, because they yield significant differences that are obtained in months. According to Iglesias-Caamaño et al. [42], at the age of 10 years, a difference of one year represents 10% of the experience of a child. Extrapolating this to the comparison between the analyzed thirds, we can find differences of 3.3%, or even greater, with the increasing difference in months in the same year of comparison. Lower EI has been related to muscles with a smaller amount of fat and a greater content of glycogen and contractile proteins [26,43]. Several studies have considered that EI in children does not change with normal growth, although such studies have been conducted on healthy, non-athlete children [44,45]. A previous study, carried out on 37 school children who practiced football (8.2 ± 0.9 years) reported significant changes in quadriceps EI after evaluation at two different time points at a 10-month interval [46]. Therefore, the EI values recorded in school children who practiced football could be an indicator of muscle quality discriminated for time points at which other physical factors of growth cannot be detected. Having maturation rate values (understood as growth in months) for the evolution of muscle quality would allow adaptation of the physical training of the athlete in a more individualized manner, which could have positive effects on the attainment of better muscle performance at younger ages.

However, the present study was limited by the small number of research units in some subgroups, which generated differences in the statistical power of the data and the exclusion of participants born in 2007 and 2011. One non-controlled variable was the training level of the participants, which could affect the performance of anaerobic power measured by vertical jump. Future studies could use other parameters for the evaluation of child development in addition to the Tanner self-report, such as the peak height velocity, which allows differentiation of those in Tanner Stage 1 based on their maturity level.

## 5. Conclusions

In a population such as the one analyzed in the present study, in a group whose age delimits a category, being born in the first months of the year results in better performance in anaerobic tests such as the vertical jump and better quantitative ultrasound measures of the quadriceps. This suggests that relative age could be an indicator of advantage in sports selection processes that involve anaerobic power, such as vertical jump, given the use of the quadriceps as the predominant muscle in the sport action.

### Practical Considerations

The selection of sport talents must use multiple tools in addition to the physical tests that are usually employed for the different sport modalities. Including the ultrasound evaluation of the muscles involved in the sport action, particularly at the school age, would make it possible to support the decision based on changes in the muscle image that may not be obvious in the sport action. Moreover, the most important aspect may be the fact that a more holistic perspective must be applied that combines all of the elements to evaluate the minor, which would avoid the consideration of immediate and hasty results that could lead to discarding talents due to the absence of vital information from an integral evaluation based on a long-term perspective.

## Figures and Tables

**Table 1:** Comparison by tertiles for anthropometric variables.

Year	Sex (Age ± SD)	Thirds	*n*	Weight (Kg)	*p*	ES			Height (cm)	*p*	ES			BMI (Kg/m^2^)	*p*	ES		
2008		CE1	3	33.2	3.8				139	1				17.18	0.23			
	Girls (*n* = 20)	CE2	6	35.25	2.95				140.5	2				18.05	1.455			
	(10.34 ± 0.26)	CE3	11	36.2	4.1	0.917	0.0092		140	5	0.704	0.0369		18.82	1.04	0.579	0.0576	
		CE1	9	46	3.2				143	4				21.29	2.77			
	Boys (*n* = 25)	CE2	7	34.2	5.1				140	2				16.72	1.72			
	(10.26 ± 0.32)	CE3	9	32.2	4.4	0.023 *	0.3141	G	135	4	0.003 *	0.4983	G	16.43	0.71	0.126	0.1723	
2009		CE1	10	36.8	4.95				141.5	2.25				17.44	1.58			
	Girls (*n* = 34)	CE2	14	32.65	3.95				140.5	5				16.12	0.885			
	(9.51 ± 0.38)	CE3	10	26.6	3.35	0.02 *	0.2358	M	131.5	3.5	0.004 **	0.3378	G	14.92	0.84	0.188	0.1013	
		CE1	16	36.95	3.65				138.25	1.75				19.19	1.65			
	Boys (*n* = 33)	CE2	5	26.4	0.2				137	4				16.35	0.97			
	(9.38 ± 0.42)	CE3	12	30.25	4.6	0.032 *	0.2158	M	136	3	0.186	0.1051		16.89	1.8	0.045 *	0.1941	M
2010		CE1	6	31.85	2.75				129	2.5				18.32	2.105			
	Girls (*n* = 16)	CE2	4	28.2	1				129.75	2.75				17.12	0.51			
	(8.44 ± 0.46)	CE3	6	29.6	2.2	0.542	0.0816		129.5	2.5	0.814	0.0275		18.35	1.3	0.476	0.0990	
		CE1	9	27.7	4.1				131	4				17.09	1.29			
	Boys (*n* = 31)	CE2	13	27.3	2.6				131	4				16.84	0.93			
	(8.28 ± 0.39)	CE3	9	28.2	3.5	0.771	0.0173		130	3	0.716	0.0222		16.43	1.41	0.671	0.0266	

*p*: * < 0.05, ** < 0.01.

**Table 2:** Comparison by tertiles in the vertical jump variables.

Year of Birth	Sex	Variable	GE1		GE2		GE3		*p* Value		E.S	
2008	Girls	CMJ	19.6	4.2	21.7	2.45	21.2	2.4	0.490		0.0751	
		PCMJ	666	286	816	52.1	856	224	0.495		0.0741	
		PCMJR	22.6	1.32	24.9	1.9	22.2	3.51	0.291		0.1298	
		PCMJDE	597	78.5	678	63.7	584	64.6	0.342		0.1129	
		PCMJDER	17	3.29	18.8	1.26	17.6	2.77	0.366		0.1058	
		PP15	157	25.8	155	15.8	143	20.4	0.826		0.0201	
	Boys	CMJ	20.4	3.1	23.8	5.4	22.9	1.7	0.503		0.0573	
		PCMJ	1338	186	912	226	629	210	0.044	*	0.2600	G
		PCMJR	28.9	2.63	29.6	2.22	22.6	2.12	0.133		0.1679	
		PCMJDE	848	78.4	755	147	630	105	0.167		0.1493	
		PCMJDER	18.5	1.79	22.6	4.03	19.6	1.39	0.394		0.0777	
		PP15	211	21.5	188	36	168	20.2	0.287		0.1040	
2009	Girls	CMJ	22.5	1.3	19.6	0.8	18.8	1.2	0.001	**	0.4131	G
		PCMJ	895	161	639	157	293	235	0.001	**	0.4069	G
		PCMJR	24.6	1.74	18.7	3.78	10.3	7.93	<0.0001	***	0.5454	G
		PCMJDE	728	100	564	59.3	495	58.6	<0.0001	***	0.4698	G
		PCMJDER	20.3	2.97	18.2	1.52	18.4	1.3	0.046	*	0.1865	M
		PP15	162	17.7	143	20.4	128	20.9	0.023	*	0.2275	M
	Boys	CMJ	18.8	2.4	19.6	0.8	18.1	2.25	0.754		0.0176	
		PCMJ	743	157	416	181	489	192	0.006	**	0.3215	G
		PCMJR	20.7	2.05	15.9	2.57	15.1	5.53	0.013	*	0.2709	G
		PCMJDE	639	69.8	514	34.3	579	76	0.030	*	0.2195	M
		PCMJDER	17	2.08	17.2	0.99	16.9	0.93	0.956		0.0028	
		PP15	161	12.7	138	6.59	145	8.45	0.025	*	0.2301	M
2010	Girls	CMJ	16.7	0.8	16.7	0.1	17.3	1.45	0.852		0.0213	
		PCMJ	427	128	302	108	474	154	0.368		0.1333	
		PCMJR	13.3	2.83	10.1	3.09	16.1	2	0.310		0.1561	
		PCMJDE	496	70.6	501	52.8	579	23.7	0.441		0.1091	
		PCMJDER	14.9	1.24	17.2	1.53	16.5	1.14	0.174		0.2329	
		PP15	122	2.62	115	10.6	117	8.13	0.284		0.1679	
	Boys	CMJ	22.9	1.7	20.4	2.5	18.8	2.1	0.158		0.1230	
		PCMJ	558	159	504	249	394	55	0.161		0.1220	
		PCMJR	24.3	3.79	17	5.27	14	1.84	0.078		0.1698	
		PCMJDE	631	144	531	119	523	68.7	0.173		0.1169	
		PCMJDER	21.2	2	17.5	3.46	18	2.1	0.202		0.1067	
		PP15	148	17.7	137	17.2	122	7.35	0.262		0.0894	

*p*: * < 0.05, ** < 0.01, *** < 0.001. CMJ: counter-movement jump (cm), PCMJ: CMJ power (w), PCMJR: relative CMJ power (w/Kg), PCMJDE: CMJ power by thrust distance, PCMJDER: relative PCMJDE (w/Kg), PP15: peak power of the best jump in 15 s (w).

**Table 3:** Comparison by tertiles in the ultrasound variables.

Year	Sex	Variable	GE1		GE2		GE3		*p* Value		E.S.	
			M	MAD	M	MAD	M	MAD				
2008	Girls	PGRF	20.7	1.24	19.9	0.59	20.9	1.32	0.371		0.1043	
		EIVIC	161	19.1	147	4.69	160	16.9	0.218		0.1604	
		PGVI	23.5	2.16	21.9	0.52	23.4	1.89	0.218		0.1604	
		Dif1C	−21	18.1	−25	6.35	−41	13.4	0.554		0.0622	
		Dif4C	−28	12.1	−31	3.69	−42	6.29	0.258		0.1425	
		EVIE	10.6	0.29	11.9	1.43	11.8	0.71	0.902		0.0108	
		ETL	29.9	1.14	29	2.68	27.1	1.93	0.970		0.0032	
		AVL	12.9	0.59	12	1.08	11.1	0.9	0.475		0.0784	
	Boys	PGRF	19.7	1.49	18.6	0.83	19.8	0.76	0.549		0.0500	
		EIVIC	146	14	140	5.7	147	7.81	0.847		0.0139	
		PGVI	21.8	1.6	21.2	0.61	22	0.88	0.788		0.0198	
		Dif1C	−22	9.15	−0.2	15.8	−14	14.2	0.228		0.1231	
		Dif4C	−0.1	13.2	−7.8	10.5	−10	11.7	0.826		0.0159	
		EVIE	14.6	3.14	11.9	1.29	11.4	0.79	0.133		0.1683	
		ETL	31.9	2.36	26.7	2.93	29.5	2	0.147		0.1595	
		AVL	13.5	2	13.8	1.75	13.4	1.32	0.694		0.0305	
2009	Girls	PGRF	20.8	1.08	20.2	0.61	19.8	0.34	0.597		0.0312	
		EIVIC	152	9.68	157	4.67	144	7.47	0.352		0.0633	
		PGVI	22.5	1.1	23.1	0.52	21.6	0.85	0.354		0.0629	
		Dif1C	−28	12.3	−30	4.47	−21	4.72	0.161		0.1107	
		Dif4C	−40	7.68	−33	6.83	−27	8.48	0.290		0.0750	
		EVIE	11.4	1.93	10.8	1.61	9.57	0.89	0.391		0.0570	
		ETL	27.3	2.39	25.6	2.43	24.2	1.71	0.514		0.0403	
		AVL	11.6	0.45	11.9	1.21	10.5	0.88	0.033	*	0.2073	M
	Boys	PGRF	20.4	0.81	19.7	0.07	19.5	1.76	0.185		0.1056	
		EIVIC	150	5.84	148	2.3	146	9.6	0.428		0.0531	
		PGVI	22.3	0.66	22	0.27	21.8	1.09	0.450		0.0499	
		Dif1C	−28	14.3	−13	6.46	−14	29.1	0.300		0.0752	
		Dif4C	−37	6.1	−38	13.5	−35	11	0.788		0.0149	
		EVIE	13.6	1.64	10.4	2.07	10.1	1.54	0.026	*	0.2283	M
		ETL	31	3.46	26.1	1.86	26	2.5	0.044	*	0.1950	M
		AVL	12.5	1.08	15.3	0.76	13.3	2.32	0.730		0.0197	
2010	Girls	PGRF	21.2	0.3	20	0.21	21.4	0.52	0.020	*	0.5189	G
		EIVIC	148	3.66	152	2.9	158	3.73	0.180		0.2284	
		PGVI	22.1	0.4	22.5	0.34	23.1	0.43	0.180		0.2284	
		Dif1C	−38	0.83	−18	1.29	−34	8.1	0.023	*	0.5032	G
		Dif4C	−39	7.8	−28	4.46	−40	6.65	0.049	*	0.4010	G
		EVIE	12.9	1.93	10.1	0.86	11.8	1.07	0.165		0.2402	
		ETL	26.9	1.11	27.4	1.46	25.1	3.96	0.855		0.0209	
		AVL	11.2	0.75	12	1.58	12.5	1.35	0.882		0.0167	
	Boys	PGRF	18.6	0.9	18.9	0.85	19.6	0.81	0.309		0.0782	
		EIVIC	134	7.5	132	7.57	143	5.24	0.033	*	0.2280	M
		PGVI	20.4	0.87	20.3	0.87	21.5	0.59	0.033	*	0.2280	M
		Dif1C	−8.4	18.7	−5.2	10.1	−16	15.3	0.316		0.0769	
		Dif4C	−31	7.89	−29	8.47	−18	9.99	0.885		0.0081	
		EVIE	11.9	1.86	10.4	2	11.4	1.07	0.443		0.0543	
		ETL	27.2	3.21	25.8	2	28.6	1.79	0.678		0.0259	
		AVL	13.7	1.42	14.5	1.92	14.6	2.32	0.872		0.0092	

*p*: * < 0.05, E.S: Effect Size, EIG: EI of fat in the anterior region of the thigh. EIRFC: corrected EI of the rectus femoris, PGRF: % fat of the rectus femoris, EIVIC: EI of the VI (anterior thigh), PGVI: % fat of the VI measured by US, Dif1C: difference between EIG and EIRFC, Dif4C: difference between EIG and EI of the VL, EVIE: VI thickness in the external region of the thigh, ETL: total thickness in the external region of the thigh, AVL: pennation angle of the vastus lateralis.

## Data Availability

All relevant data are within the study.
